# Enhancing Lifestyle Counselling Targeted at Weight Management for Patients on Antipsychotics in a Community Mental Health Team: A Quality Improvement Project

**DOI:** 10.1192/bjo.2025.10381

**Published:** 2025-06-20

**Authors:** Madhulika Joglekar, Keya Patel, Sungjun Park, Peeyush Roy, Olubunmi Olure

**Affiliations:** 1 GKT School of Medical Education, King’s College London, London, United Kingdom; 2 Kent and Medway NHS and Social Care Partnership Trust, Kent, United Kingdom.; 3 Royal College of Psychiatrists, London, United Kingdom

## Abstract

**Aims:** The association between antipsychotic medication and weight gain is widely recognised. The Royal College of Psychiatrists’ Positive Cardiometabolic Health Resource recommends lifestyle counselling as per the National Health Service Eat Well and Live Well guides, and the United Kingdom Chief Medical Officers’ Guidance on physical activity, to promote positive lifestyle behaviours and a healthy BMI in patients with psychosis. Owing to a lack of educational resources for healthcare professionals, patients at a Community Mental Health Team in Kent were not exposed to thorough lifestyle counselling. This project aimed to improve staff awareness and confidence with respect to the recommended dietary and physical activity counselling guidelines by 80% within a four-month period.

**Methods:** This project was structured using two Plan, Do, Study, Act (PDSA) cycles. Baseline data, sought through an initial survey (S1) consisting of Likert scale and multiple-choice questions (MCQs) assessed staff perceptions of their awareness and confidence with respect to guidelines. PDSA1 involved the display of informational posters with key lifestyle recommendations in all consultation rooms and shared areas. Upon data analysis and participant feedback, PDSA2 involved an educational session delivered to staff, covering the recommended lifestyle guidance. Two identical surveys (S2 and S3) were distributed after each intervention to assess any changes.

**Results:** S1 revealed considerable variation around the average staff awareness and confidence level (M=5.32, SD=3.461). Although this dropped in S2 (M=4.7, SD=3.303), an increase was observed in S3 (M=4.96, SD=2.166). The initial decrease could be explained by staff overestimation of their awareness and confidence at baseline, which may have been realised once poster resources were introduced. The following increase in mean awareness and confidence suggests a positive impact of the teaching session delivered within PDSA2.

A similar trend was observed in the MCQs. The mean percentage of correct MCQ answers in S1 was 36.0%. Despite an initial decrease to 26.0% in S2, this value increased to 38.9% in S3. This shows an improvement in the knowledge of staff and ability to recall specific lifestyle guidance.

**Conclusion:** Despite not achieving our initial aim, active teaching was more effective in improving staff awareness and confidence levels regarding patient lifestyle counselling, compared with passive methods such as posters. The delivery of educational sessions, coupled with the provision of supplemental informational resources for patient-facing staff, could further improve awareness and confidence by allowing healthcare professionals to apply the recommended guidance in consultations, and ensure sustainability of their knowledge.

